# Robotic repair of vesicovaginal fistula - initial experience

**DOI:** 10.1590/S1677-5538.IBJU.2014.0629

**Published:** 2016

**Authors:** Ankush Jairath, S.B Sudharsan, Shashikant Mishra, Arvind Ganpule, Ravindra Sabnis, Mahesh Desai

**Affiliations:** 1 Department of Urology, Muljibhai Patel Urological Hospital (MPUH). Nadiad, India

## Abstract

**Objective:**

The most common acquired fistula of the urinary tract is Vesicovaginal fistulae (VVF) (1) posing social stigmata for the patient as well as a surgical challenge for the urologist. Here we present our initial experience with Robotic assisted laparoscopic repair of VVF, its safety and efficacy.

**Materials and Methods:**

Seven out of eight fistulas were post hysterectomy; five had undergone abdominal while two had laparoscopic hysterectomy while one was due to prolonged labour. Two had associated ureteric injury. All underwent robotic assisted laparoscopic trans abdominal extravesical approach. Three 8 mm ports for robotic arms, one 12 mm port for camera and another 12 mm for assistant were used in a fan shaped manner. All had preoperative ureteric catheter placed. Bladder was closed in two layers and vagina in one layer. Omental flap placed in all cases except two where it was not possible. Drain and per urethral catheter placed in all cases. Double J stents were placed in two cases requiring ureteric implantation additionally.

**Results:**

The mean age of presentation was 39.25 years (26-47 range) with mean BMI being 26.25 kg/m2 (21-32 range). Mean duration between insult and repair was 9.37 months (3-24 months). Only in single case there was history of previous repair attempt. On cystoscopy four had supratrigonal VVF and four were trigonal with mean size of 13.37 mm (7-20 mm). Mean operative time was 117.5 minutes (90-150). There were no intraoperative/postoperative complications or need for open conversion. Mean haemoglobin drop was 1.4 gm/dL (0.3-2 gm). Drain was removed once 24-48 hours output is negligible. One patient had post-operative urinary leak at 2 weeks which ceased with continuation of catheterisation for another 2 weeks. Catheter was removed after voiding cystourethrogram showed no leak at 2-3 weeks postoperatively. Mean duration of drain was 3.75 days (3-5) and per urethral catheterisation (which was removed after voiding cystourethrography) was 15.75 days (9-28). Mean hospital stay was 6.62 days (4-14). Post-operative bladder capacity was 324.28 cc (280-350) on voiding diary. Follow up ranged from 3-9 months. At 3 months of follow-up, these patients continued to void normally and there was no evidence of recurrence of VVF.

**Conclusion:**

Robotic repair of VVF is safe and feasible and has additional advantages in the form of precise suturing under 3D vision and certainly a more striking and effective option especially in complex VVF repair associated with ureteric injuries (2).

## ARTICLE INFO

Available at: http://www.intbrazjurol.com.br/video-section/video-library/jairath_168_169/

Int braz J urol. 2016; 42 (Video #1): 168-9

## References

[1] Gerber GS, Schoenberg HW (1993). Female urinary tract fistulas. J Urol.

[2] Sundaram BM, Kalidasan G, Hemal AK (2006). Robotic repair of vesicovaginal fistula: case series of five patients. Urology.

