# Supplementary material for: Robotic repair of vesicovaginal fistula - initial experience
Source: Int Braz J Urol. 2016 Jan-Feb;42(1):168–9. doi: 10.1590/S1677-5538.IBJU.2014.0629 (PMC4811244; doi:10.1590/S1677-5538.IBJU.2014.0629)
Supplement: Related texts

# Reply: EDITORIAL COMMENT

These authors have reported one of the largest series of robotic assisted laparoscopic repair of vesico-vaginal fistula (VVF). They have demonstrated that robotic repair of VVF is associated with minimal morbidity with excellent 3 month follow up outcomes. As urologists have advanced their robotic expertise, they are now able to tackle the more difficult cases of pelvic reconstruction. VVF can represent a challenge due to both etiology and variability in anatomy. The authors are correct in their commentary that the robotic platform with its 3D vision system and precise instrumentation in the pelvis allow it to be an effective option when considering VVF repair. While urologists can add this to their robotic armamentarium, I would caution that such cases should be only attempted by seasoned robotic surgeons.

*Trushar Patel, MD* *Assistant Professor of Urology* *Director of Robotic Urologic Surgery, Florida Hospital Tampa* *Morsani College of Medicine* *University of South Florida, USA* *E-mail: trushar.patelmd@gmail.com*
